# Ways to make artificial intelligence work for healthcare professionals: correspondence

**DOI:** 10.1017/ash.2024.85

**Published:** 2024-06-04

**Authors:** Hinpetch Daungsupawong, Viroj Wiwanitkit

**Affiliations:** 1 Private Academic Consultant, Phonhong, Lao People’s Democratic Republic, Phonhong, Vientiane Province, Laos; ^2^Department of Research Analytics, Saveetha Dental College and Hospitals, Saveetha Institute of Medical and Technical Sciences Saveetha University, Kanchipuram, Tamil Nadu, India

Dear editor, we hereby discuss the publication “All aboard the ChatGPT steamroller: Top 10 ways to make artificial intelligence work for healthcare professionals.”^1^ Non has already discussed several of the restrictions identified. The purpose of this letter is to underline and highlight some additional limitations of Large Langauge Model (LLM) that were not previously mentioned by the original author. While there are a number of possible advantages to ChatGPT integration in medicine, there are also a number of disadvantages and issues that need to be resolved. Although ChatGPT is a language model that has been extensively trained on data, it might not have the medical background or context required to deliver accurate and trustworthy results. Medical practitioners depend on evidence-based procedures, thus, there’s a chance ChatGPT will give inaccurate or misleading information, which could result in medical mistakes.^2^ Because ChatGPT relies on its training data to function, privacy and bias problems are brought up ethically. The artificial intelligence (AI) chatbot can unintentionally reinforce prejudice or discrimination in healthcare encounters if it is not built using a broad and representative dataset. Furthermore, the protection of patient data has to be a top concern, and stringent privacy regulations must be followed while utilizing AI systems.^3^

To guarantee improved patient results, it’s critical to find a balance between technology and interpersonal communication. AI chatbots, such as ChatGPT should be subject to human oversight by medical practitioners in the future. This would entail incorporating a human-in-the-loop system in which medical professionals oversee and select the chatbot algorithms to guarantee correct and trustworthy responses.^4^

The chatbot’s capacity to provide precise medical advice and expertise can be improved by creating specific AI models that have undergone thorough training using clinical guidelines, medical literature, and expert viewpoints. Enhancing the model’s domain-specific competency can be achieved by training it solely on healthcare-related data. Artificial intelligence chatbots in the medical field ought to be built to pick up on interactions and take patient and professional input into account. In addition to the well-known healthcare-tailored LLM, there are numerous healthcare-focused LLMs in development that have received little attention, such as ClinicalBERT and Med-PaLM 2.^5,6^ ClinicalBERT was recently presented to pre-train contextualized word representation models utilizing bidirectional transformers, significantly improving the accuracy of several natural language processing tasks.^7^ Med-PaLM 2 was developed specifically for biomedical research, such as assessing gene-phenotype connections and generating fresh ideas, which can aid in genetic discoveries.^6^

In addition to being useful in general healthcare, specialized LLMs in infectious diseases and antibiotic stewardship are intriguing. The potential application in clinical consulting is emphasized,^8^ raising some concerns about the direction cognitive specialties are taking. Nevertheless, there are currently issues with LLMs that make safe clinical deployment in specialist consultations impossible. These issues include a tendency to recapitulate biases, frequent confabulations, a lack of contextual awareness that is essential for complex diagnostic and treatment plans, and mysterious and unexplainable training data and methods.^8^ By using an iterative process, the model will be improved over time, and its applicability and dependability in actual medical situations will be guaranteed. In the end, it’s critical to remember that the artificial intelligence system’s user ultimately decides whether or not to adhere to a just and moral standard.^4^

## Data Availability

There is no new data generated.
